# 4-[(9-Ethyl-9*H*-carbazol-3-yl)methyl­idene­amino]-1,5-dimethyl-2-phenyl-1*H*-pyrazol-3(2*H*)-one

**DOI:** 10.1107/S1600536810023779

**Published:** 2010-06-26

**Authors:** Abdullah M. Asiri, Salman A. Khan, Kong Wai Tan, Seik Weng Ng

**Affiliations:** a Chemistry Department, Faculty of Science, King Abdul Aziz University, PO Box 80203, Jeddah 21589, Saudi Arabia; b Department of Chemistry, University of Malaya, 50603 Kuala Lumpur, Malaysia

## Abstract

The imino–carbon double bond in the title Schiff base, C_26_H_24_N_4_O, has an *E* configuration. The 13-membered carbazolyl fused-ring (r.m.s. deviation = 0.056 Å) is nearly coplanar with five-membered pyrazole ring (r.m.s. deviation = 0.036 Å) [dihedral angle between the two systems = 10.4 (1)°]; the phenyl substituent is twisted by 51.1 (1)° with respect to the five-membered ring.

## Related literature

For background to this class of Schiff bases, see: Montalvo-González & Ariza-Castolo (2003 ▶).
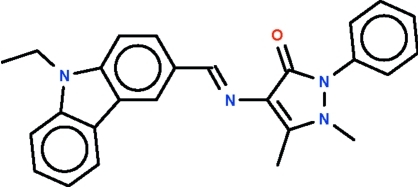


## Experimental

### 

#### Crystal data

C_26_H_24_N_4_O
*M* *_r_* = 408.49
Monoclinic, 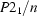

*a* = 10.4458 (6) Å
*b* = 18.2674 (11) Å
*c* = 10.8989 (6) Å
β = 96.127 (1)°
*V* = 2067.8 (2) Å^3^
*Z* = 4
Mo *K*α radiation
μ = 0.08 mm^−1^
*T* = 100 K
0.25 × 0.20 × 0.20 mm

#### Data collection

Bruker SMART APEX diffractometer
19772 measured reflections
4756 independent reflections
4000 reflections with *I* > 2σ(*I*)
*R*_int_ = 0.031

#### Refinement

*R*[*F* ^2^ > 2σ(*F* ^2^)] = 0.038
*wR*(*F* ^2^) = 0.104
*S* = 1.02
4756 reflections
283 parameters
H-atom parameters constrained
Δρ_max_ = 0.24 e Å^−3^
Δρ_min_ = −0.25 e Å^−3^

### 

Data collection: *APEX2* (Bruker, 2009 ▶); cell refinement: *SAINT* (Bruker, 2009 ▶); data reduction: *SAINT*; program(s) used to solve structure: *SHELXS97* (Sheldrick, 2008 ▶); program(s) used to refine structure: *SHELXL97* (Sheldrick, 2008 ▶); molecular graphics: *X-SEED* (Barbour, 2001 ▶); software used to prepare material for publication: *publCIF* (Westrip, 2010 ▶).

## Supplementary Material

Crystal structure: contains datablocks global, I. DOI: 10.1107/S1600536810023779/jh2169sup1.cif

Structure factors: contains datablocks I. DOI: 10.1107/S1600536810023779/jh2169Isup2.hkl

Additional supplementary materials:  crystallographic information; 3D view; checkCIF report

## Figures and Tables

**Fig. 1.**
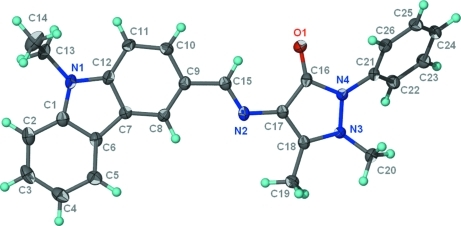
Thermal ellipsoid plot (Barbour, 2001) of C26H24N4O at the 70% probability level; hydrogen atoms are drawn as spheres of arbitrary radius.

## supplementary crystallographic information

### Comment

4-Aminoantipyrine (4-amino-1,2-dihydro-1,5-dimethyl-2-phenyl-3*H*-pyrazol-3-one) possesses a pyrazolone unit, a feature that is important to the design of non-steroidal anti-inflammatory chemicals. Its amino group group allows the chemical to condense with aromatic aldehydes to yield Schiff bases. The crystal structures of a large number of such Schiff bases have been reported. For the Schiff base derived from the benzaldehyde homolog, the phenyl and pyrazoly rings are nearly coplanar (Montalvo-González & Ariza-Castolo, 2003). In the title carbazole-aldehyde analog (Scheme I, Fig. 1), the 13-membered carbazolyl fused-ring is nearly coplanar with 5-membered pyrazolyl ring [dihedral angle between the two systems 10.4 (1) °]. The phenyl substituent is twisted by 51.1 (1) ° with respect to the 5-membered ring.

### Experimental

9-Ethylcarbazole-3-carboxaldehyde (0.50 g, 2.2 mmol) and 4-aminoantipyrine (0.45 g, 2.2 mmol) here heated in methanol (15 ml) for 5 h to afford a light yellow precipitate. The solid material was collected and recrystallized from methanol.

### Refinement

Carbon-bound H-atoms were placed in calculated positions [C–H 0.95 to 0.98 Å, *U*(H) 1.2 to 1.5*U*_eq_(C)] and were included in the refinement in the riding model approximation.

### Crystal data

**Table:**

C_26_H_24_N_4_O	*F*(000) = 864
*M**_r_* = 408.49	*D*_x_ = 1.312 Mg m^−^^3^
Monoclinic, *P*2_1_/*n*	Mo *K*α radiation, λ = 0.71073 Å
Hall symbol: -P 2yn	Cell parameters from 7258 reflections
*a* = 10.4458 (6) Å	θ = 2.2–28.3°
*b* = 18.2674 (11) Å	µ = 0.08 mm^−^^1^
*c* = 10.8989 (6) Å	*T* = 100 K
β = 96.127 (1)°	Prism, yellow
*V* = 2067.8 (2) Å^3^	0.25 × 0.20 × 0.20 mm
*Z* = 4	

### Data collection

**Table:**

Bruker SMART APEX diffractometer	4000 reflections with *I* > 2σ(*I*)
Radiation source: fine-focus sealed tube	*R*_int_ = 0.031
graphite	θ_max_ = 27.5°, θ_min_ = 2.2°
ω scans	*h* = −13→13
19772 measured reflections	*k* = −23→23
4756 independent reflections	*l* = −14→14

### Refinement

**Table:**

Refinement on *F*^2^	Primary atom site location: structure-invariant direct methods
Least-squares matrix: full	Secondary atom site location: difference Fourier map
*R*[*F*^2^ > 2σ(*F*^2^)] = 0.038	Hydrogen site location: inferred from neighbouring sites
*wR*(*F*^2^) = 0.104	H-atom parameters constrained
*S* = 1.02	*w* = 1/[σ^2^(*F*_o_^2^) + (0.0525*P*)^2^ + 0.720*P*] where *P* = (*F*_o_^2^ + 2*F*_c_^2^)/3
4756 reflections	(Δ/σ)_max_ < 0.001
283 parameters	Δρ_max_ = 0.24 e Å^−^^3^
0 restraints	Δρ_min_ = −0.25 e Å^−^^3^

### Special details

**Table:**

Geometry. All e.s.d.'s (except the e.s.d. in the dihedral angle between two l.s. planes) are estimated using the full covariance matrix. The cell e.s.d.'s are taken into account individually in the estimation of e.s.d.'s in distances, angles and torsion angles; correlations between e.s.d.'s in cell parameters are only used when they are defined by crystal symmetry. An approximate (isotropic) treatment of cell e.s.d.'s is used for estimating e.s.d.'s involving l.s. planes.

### Fractional atomic coordinates and isotropic or equivalent isotropic displacement parameters (Å^2^)

**Table:**

	*x*	*y*	*z*	*U*_iso_*/*U*_eq_	
O1	0.60645 (8)	0.54578 (4)	0.40022 (8)	0.02038 (19)	
N1	1.35714 (9)	0.68186 (6)	0.65745 (9)	0.0206 (2)	
N2	0.75630 (9)	0.69353 (6)	0.47222 (9)	0.0175 (2)	
N3	0.43648 (9)	0.70479 (5)	0.32066 (9)	0.0178 (2)	
N4	0.44409 (9)	0.62867 (5)	0.33728 (9)	0.0179 (2)	
C1	1.36337 (11)	0.75748 (7)	0.66819 (11)	0.0196 (2)	
C2	1.46850 (12)	0.80129 (8)	0.70917 (12)	0.0250 (3)	
H2	1.5508	0.7805	0.7330	0.030*	
C3	1.44906 (13)	0.87620 (8)	0.71402 (12)	0.0269 (3)	
H3	1.5192	0.9072	0.7419	0.032*	
C4	1.32833 (13)	0.90704 (7)	0.67875 (11)	0.0255 (3)	
H4	1.3177	0.9586	0.6833	0.031*	
C5	1.22368 (12)	0.86349 (7)	0.63713 (11)	0.0213 (3)	
H5	1.1420	0.8848	0.6126	0.026*	
C6	1.24060 (11)	0.78774 (7)	0.63197 (10)	0.0178 (2)	
C7	1.15504 (11)	0.72649 (6)	0.60145 (10)	0.0165 (2)	
C8	1.02461 (11)	0.72027 (6)	0.56055 (10)	0.0167 (2)	
H8	0.9737	0.7629	0.5434	0.020*	
C9	0.96945 (11)	0.65098 (6)	0.54505 (10)	0.0172 (2)	
C10	1.04661 (12)	0.58849 (6)	0.57261 (11)	0.0193 (2)	
H10	1.0070	0.5416	0.5660	0.023*	
C11	1.17744 (11)	0.59244 (6)	0.60877 (11)	0.0196 (2)	
H11	1.2283	0.5496	0.6241	0.023*	
C12	1.23104 (11)	0.66235 (7)	0.62169 (10)	0.0179 (2)	
C13	1.46091 (11)	0.63045 (7)	0.69520 (11)	0.0223 (3)	
H13A	1.5445	0.6535	0.6832	0.027*	
H13B	1.4509	0.5864	0.6420	0.027*	
C14	1.46196 (15)	0.60749 (9)	0.82923 (13)	0.0357 (3)	
H14A	1.5318	0.5723	0.8501	0.054*	
H14B	1.3794	0.5847	0.8416	0.054*	
H14C	1.4754	0.6506	0.8825	0.054*	
C15	0.83444 (11)	0.63985 (6)	0.49658 (10)	0.0175 (2)	
H15	0.8037	0.5913	0.4827	0.021*	
C16	0.56810 (11)	0.60962 (6)	0.38971 (10)	0.0165 (2)	
C17	0.63052 (11)	0.67924 (6)	0.42004 (10)	0.0163 (2)	
C18	0.54672 (11)	0.73382 (6)	0.37959 (10)	0.0169 (2)	
C19	0.56536 (12)	0.81422 (6)	0.39240 (11)	0.0208 (3)	
H19A	0.5360	0.8382	0.3139	0.031*	
H19B	0.6569	0.8248	0.4147	0.031*	
H19C	0.5155	0.8327	0.4571	0.031*	
C20	0.31008 (11)	0.73921 (7)	0.31473 (12)	0.0221 (3)	
H20A	0.3203	0.7914	0.3339	0.033*	
H20B	0.2596	0.7160	0.3748	0.033*	
H20C	0.2654	0.7334	0.2316	0.033*	
C21	0.35842 (11)	0.58088 (6)	0.26541 (10)	0.0170 (2)	
C22	0.31925 (12)	0.59525 (7)	0.14157 (11)	0.0207 (3)	
H22	0.3482	0.6380	0.1034	0.025*	
C23	0.23735 (12)	0.54634 (7)	0.07451 (11)	0.0217 (3)	
H23	0.2100	0.5560	−0.0099	0.026*	
C24	0.19515 (11)	0.48364 (7)	0.12940 (11)	0.0209 (3)	
H24	0.1394	0.4503	0.0830	0.025*	
C25	0.23514 (12)	0.47000 (7)	0.25284 (11)	0.0208 (2)	
H25	0.2067	0.4269	0.2906	0.025*	
C26	0.31609 (11)	0.51841 (6)	0.32186 (11)	0.0186 (2)	
H26	0.3422	0.5090	0.4066	0.022*	

### Atomic displacement parameters (Å^2^)

**Table:**

	*U*^11^	*U*^22^	*U*^33^	*U*^12^	*U*^13^	*U*^23^
O1	0.0190 (4)	0.0166 (4)	0.0248 (4)	0.0015 (3)	−0.0012 (3)	0.0022 (3)
N1	0.0129 (5)	0.0230 (5)	0.0254 (5)	0.0016 (4)	−0.0005 (4)	−0.0015 (4)
N2	0.0141 (5)	0.0215 (5)	0.0164 (4)	−0.0007 (4)	−0.0006 (4)	−0.0016 (4)
N3	0.0157 (5)	0.0142 (5)	0.0227 (5)	0.0007 (4)	−0.0022 (4)	0.0005 (4)
N4	0.0160 (5)	0.0145 (5)	0.0222 (5)	−0.0003 (4)	−0.0030 (4)	0.0019 (4)
C1	0.0169 (6)	0.0236 (6)	0.0187 (5)	−0.0015 (5)	0.0040 (4)	−0.0004 (4)
C2	0.0173 (6)	0.0327 (7)	0.0250 (6)	−0.0050 (5)	0.0031 (5)	−0.0012 (5)
C3	0.0263 (6)	0.0318 (7)	0.0229 (6)	−0.0139 (5)	0.0040 (5)	−0.0009 (5)
C4	0.0346 (7)	0.0208 (6)	0.0214 (6)	−0.0079 (5)	0.0043 (5)	0.0010 (5)
C5	0.0249 (6)	0.0203 (6)	0.0186 (5)	−0.0017 (5)	0.0024 (5)	0.0018 (4)
C6	0.0166 (5)	0.0219 (6)	0.0153 (5)	−0.0020 (4)	0.0029 (4)	0.0001 (4)
C7	0.0174 (6)	0.0171 (5)	0.0153 (5)	0.0005 (4)	0.0027 (4)	0.0000 (4)
C8	0.0162 (5)	0.0178 (5)	0.0159 (5)	0.0017 (4)	0.0009 (4)	0.0002 (4)
C9	0.0155 (5)	0.0204 (6)	0.0154 (5)	0.0008 (4)	0.0009 (4)	−0.0015 (4)
C10	0.0207 (6)	0.0167 (6)	0.0201 (5)	−0.0003 (4)	0.0006 (4)	−0.0033 (4)
C11	0.0190 (6)	0.0175 (6)	0.0218 (6)	0.0042 (4)	0.0002 (4)	−0.0023 (4)
C12	0.0142 (5)	0.0222 (6)	0.0173 (5)	0.0018 (4)	0.0009 (4)	−0.0012 (4)
C13	0.0141 (5)	0.0283 (6)	0.0243 (6)	0.0055 (5)	0.0010 (4)	−0.0021 (5)
C14	0.0337 (8)	0.0478 (9)	0.0254 (7)	0.0170 (7)	0.0021 (6)	0.0019 (6)
C15	0.0174 (5)	0.0190 (6)	0.0159 (5)	−0.0016 (4)	0.0004 (4)	−0.0016 (4)
C16	0.0142 (5)	0.0212 (6)	0.0138 (5)	−0.0005 (4)	0.0002 (4)	0.0017 (4)
C17	0.0157 (5)	0.0185 (6)	0.0147 (5)	−0.0008 (4)	0.0011 (4)	−0.0008 (4)
C18	0.0157 (5)	0.0198 (6)	0.0149 (5)	−0.0013 (4)	0.0009 (4)	−0.0016 (4)
C19	0.0218 (6)	0.0172 (6)	0.0226 (6)	0.0001 (5)	−0.0017 (5)	−0.0023 (4)
C20	0.0167 (6)	0.0225 (6)	0.0265 (6)	0.0028 (5)	−0.0001 (5)	0.0001 (5)
C21	0.0128 (5)	0.0174 (5)	0.0202 (5)	−0.0003 (4)	0.0000 (4)	−0.0006 (4)
C22	0.0215 (6)	0.0188 (6)	0.0215 (6)	−0.0037 (5)	0.0003 (5)	0.0039 (4)
C23	0.0238 (6)	0.0216 (6)	0.0188 (6)	−0.0006 (5)	−0.0024 (5)	0.0012 (5)
C24	0.0182 (6)	0.0187 (6)	0.0251 (6)	−0.0020 (5)	−0.0001 (5)	−0.0036 (5)
C25	0.0203 (6)	0.0168 (6)	0.0256 (6)	−0.0017 (5)	0.0043 (5)	0.0025 (5)
C26	0.0172 (5)	0.0199 (6)	0.0185 (5)	0.0017 (4)	0.0020 (4)	0.0021 (4)

### Geometric parameters (Å, °)

**Table:**

O1—C16	1.2344 (14)	C11—C12	1.3956 (17)
N1—C12	1.3799 (15)	C11—H11	0.9500
N1—C1	1.3873 (16)	C13—C14	1.5187 (18)
N1—C13	1.4598 (15)	C13—H13A	0.9900
N2—C15	1.2853 (15)	C13—H13B	0.9900
N2—C17	1.3994 (14)	C14—H14A	0.9800
N3—C18	1.3646 (14)	C14—H14B	0.9800
N3—N4	1.4034 (13)	C14—H14C	0.9800
N3—C20	1.4577 (15)	C15—H15	0.9500
N4—C16	1.4028 (14)	C16—C17	1.4511 (16)
N4—C21	1.4237 (14)	C17—C18	1.3682 (16)
C1—C2	1.3933 (17)	C18—C19	1.4861 (16)
C1—C6	1.4134 (16)	C19—H19A	0.9800
C2—C3	1.385 (2)	C19—H19B	0.9800
C2—H2	0.9500	C19—H19C	0.9800
C3—C4	1.397 (2)	C20—H20A	0.9800
C3—H3	0.9500	C20—H20B	0.9800
C4—C5	1.3884 (17)	C20—H20C	0.9800
C4—H4	0.9500	C21—C26	1.3907 (16)
C5—C6	1.3969 (17)	C21—C22	1.3929 (16)
C5—H5	0.9500	C22—C23	1.3895 (16)
C6—C7	1.4484 (16)	C22—H22	0.9500
C7—C8	1.3922 (16)	C23—C24	1.3857 (17)
C7—C12	1.4192 (16)	C23—H23	0.9500
C8—C9	1.3935 (16)	C24—C25	1.3880 (17)
C8—H8	0.9500	C24—H24	0.9500
C9—C10	1.4112 (16)	C25—C26	1.3881 (16)
C9—C15	1.4664 (15)	C25—H25	0.9500
C10—C11	1.3832 (16)	C26—H26	0.9500
C10—H10	0.9500		
			
C12—N1—C1	108.40 (10)	C14—C13—H13B	109.2
C12—N1—C13	124.84 (10)	H13A—C13—H13B	107.9
C1—N1—C13	126.16 (10)	C13—C14—H14A	109.5
C15—N2—C17	119.32 (10)	C13—C14—H14B	109.5
C18—N3—N4	106.92 (9)	H14A—C14—H14B	109.5
C18—N3—C20	124.62 (10)	C13—C14—H14C	109.5
N4—N3—C20	118.15 (9)	H14A—C14—H14C	109.5
C16—N4—N3	109.60 (9)	H14B—C14—H14C	109.5
C16—N4—C21	124.82 (10)	N2—C15—C9	122.27 (11)
N3—N4—C21	120.86 (9)	N2—C15—H15	118.9
N1—C1—C2	128.85 (11)	C9—C15—H15	118.9
N1—C1—C6	109.44 (10)	O1—C16—N4	123.27 (10)
C2—C1—C6	121.70 (12)	O1—C16—C17	132.33 (11)
C3—C2—C1	117.77 (12)	N4—C16—C17	104.38 (9)
C3—C2—H2	121.1	C18—C17—N2	122.39 (10)
C1—C2—H2	121.1	C18—C17—C16	107.99 (10)
C2—C3—C4	121.29 (12)	N2—C17—C16	129.46 (10)
C2—C3—H3	119.4	N3—C18—C17	110.31 (10)
C4—C3—H3	119.4	N3—C18—C19	121.59 (10)
C5—C4—C3	121.00 (12)	C17—C18—C19	128.09 (10)
C5—C4—H4	119.5	C18—C19—H19A	109.5
C3—C4—H4	119.5	C18—C19—H19B	109.5
C4—C5—C6	118.82 (12)	H19A—C19—H19B	109.5
C4—C5—H5	120.6	C18—C19—H19C	109.5
C6—C5—H5	120.6	H19A—C19—H19C	109.5
C5—C6—C1	119.41 (11)	H19B—C19—H19C	109.5
C5—C6—C7	134.17 (11)	N3—C20—H20A	109.5
C1—C6—C7	106.34 (10)	N3—C20—H20B	109.5
C8—C7—C12	119.64 (10)	H20A—C20—H20B	109.5
C8—C7—C6	134.06 (11)	N3—C20—H20C	109.5
C12—C7—C6	106.30 (10)	H20A—C20—H20C	109.5
C7—C8—C9	119.39 (11)	H20B—C20—H20C	109.5
C7—C8—H8	120.3	C26—C21—C22	120.59 (11)
C9—C8—H8	120.3	C26—C21—N4	118.08 (10)
C8—C9—C10	119.31 (10)	C22—C21—N4	121.33 (10)
C8—C9—C15	122.61 (10)	C23—C22—C21	119.29 (11)
C10—C9—C15	118.04 (10)	C23—C22—H22	120.4
C11—C10—C9	122.89 (11)	C21—C22—H22	120.4
C11—C10—H10	118.6	C24—C23—C22	120.71 (11)
C9—C10—H10	118.6	C24—C23—H23	119.6
C10—C11—C12	116.75 (11)	C22—C23—H23	119.6
C10—C11—H11	121.6	C23—C24—C25	119.35 (11)
C12—C11—H11	121.6	C23—C24—H24	120.3
N1—C12—C11	128.71 (11)	C25—C24—H24	120.3
N1—C12—C7	109.39 (10)	C26—C25—C24	120.92 (11)
C11—C12—C7	121.87 (10)	C26—C25—H25	119.5
N1—C13—C14	112.18 (10)	C24—C25—H25	119.5
N1—C13—H13A	109.2	C25—C26—C21	119.14 (11)
C14—C13—H13A	109.2	C25—C26—H26	120.4
N1—C13—H13B	109.2	C21—C26—H26	120.4
			
C18—N3—N4—C16	−9.38 (12)	C8—C7—C12—C11	−3.85 (17)
C20—N3—N4—C16	−156.12 (10)	C6—C7—C12—C11	175.87 (11)
C18—N3—N4—C21	−166.14 (10)	C12—N1—C13—C14	−81.94 (15)
C20—N3—N4—C21	47.12 (15)	C1—N1—C13—C14	88.12 (15)
C12—N1—C1—C2	175.55 (12)	C17—N2—C15—C9	176.64 (10)
C13—N1—C1—C2	4.1 (2)	C8—C9—C15—N2	−4.26 (18)
C12—N1—C1—C6	−3.37 (13)	C10—C9—C15—N2	178.02 (11)
C13—N1—C1—C6	−174.79 (11)	N3—N4—C16—O1	−170.71 (10)
N1—C1—C2—C3	−178.53 (12)	C21—N4—C16—O1	−15.08 (18)
C6—C1—C2—C3	0.28 (18)	N3—N4—C16—C17	7.67 (12)
C1—C2—C3—C4	−0.18 (19)	C21—N4—C16—C17	163.30 (10)
C2—C3—C4—C5	−0.24 (19)	C15—N2—C17—C18	−172.81 (11)
C3—C4—C5—C6	0.56 (18)	C15—N2—C17—C16	1.92 (18)
C4—C5—C6—C1	−0.46 (17)	O1—C16—C17—C18	174.95 (12)
C4—C5—C6—C7	175.81 (12)	N4—C16—C17—C18	−3.22 (12)
N1—C1—C6—C5	179.06 (10)	O1—C16—C17—N2	−0.4 (2)
C2—C1—C6—C5	0.04 (17)	N4—C16—C17—N2	−178.54 (11)
N1—C1—C6—C7	1.84 (13)	N4—N3—C18—C17	7.25 (13)
C2—C1—C6—C7	−177.17 (11)	C20—N3—C18—C17	151.26 (11)
C5—C6—C7—C8	3.4 (2)	N4—N3—C18—C19	−173.40 (10)
C1—C6—C7—C8	179.98 (12)	C20—N3—C18—C19	−29.39 (17)
C5—C6—C7—C12	−176.29 (13)	N2—C17—C18—N3	173.21 (10)
C1—C6—C7—C12	0.32 (12)	C16—C17—C18—N3	−2.51 (13)
C12—C7—C8—C9	2.68 (17)	N2—C17—C18—C19	−6.09 (19)
C6—C7—C8—C9	−176.94 (12)	C16—C17—C18—C19	178.19 (11)
C7—C8—C9—C10	0.78 (16)	C16—N4—C21—C26	62.98 (15)
C7—C8—C9—C15	−176.91 (10)	N3—N4—C21—C26	−143.95 (11)
C8—C9—C10—C11	−3.41 (17)	C16—N4—C21—C22	−116.02 (13)
C15—C9—C10—C11	174.39 (11)	N3—N4—C21—C22	37.05 (16)
C9—C10—C11—C12	2.29 (18)	C26—C21—C22—C23	−0.13 (18)
C1—N1—C12—C11	−174.53 (12)	N4—C21—C22—C23	178.84 (11)
C13—N1—C12—C11	−2.97 (19)	C21—C22—C23—C24	−0.31 (19)
C1—N1—C12—C7	3.58 (13)	C22—C23—C24—C25	0.22 (19)
C13—N1—C12—C7	175.14 (10)	C23—C24—C25—C26	0.31 (18)
C10—C11—C12—N1	179.23 (11)	C24—C25—C26—C21	−0.74 (18)
C10—C11—C12—C7	1.34 (17)	C22—C21—C26—C25	0.65 (18)
C8—C7—C12—N1	177.90 (10)	N4—C21—C26—C25	−178.36 (10)
C6—C7—C12—N1	−2.39 (13)		
